# Structural insights into ligand-induced CARD assembly in inflammatory caspases

**DOI:** 10.21203/rs.3.rs-10541886/v1

**Published:** 2026-09-09

**Authors:** Zhongyi Jiang, Jun Bae Park, Youn Jung Choi, Stephanie Kim, Masaru Miyagi, Stephanie Leon Quinonez, Jae U. Jung

**Affiliations:** 1 Department of Microbial Sciences in Health, Cleveland Clinic Research, Cleveland Clinic, Cleveland, Ohio, USA; 2 Global Center for Pathogen and Human Health Research, Cleveland Clinic Research, Cleveland Clinic, Cleveland, Ohio, USA; 3 Department of Molecular Medicine, Cleveland Clinic Lerner College of Medicine, Case Western Reserve University, Cleveland, Ohio, USA; 4 Kao Autoimmunity Institute, Department of Medicine, Cedars-Sinai Medical Center, Los Angeles, California, USA; 5 Department of Biomedical Sciences, Cedars-Sinai Medical Center, Los Angeles, California, USA; 6 Department of Pharmacology, Case Western Reserve University, Cleveland, Ohio, USA

## Abstract

Non-canonical inflammatory caspases detect cytosolic lipopolysaccharide (LPS) through its caspase activation and recruitment domain (CARD), yet how ligand binding drives higher-order CARD assembly remains poorly understood. Here, we identify CBM7, a minimal SERPINB1-derived peptide, that induces caspase-4 CARD oligomerization. CBM7 promotes formation of a previously unrecognized flexible filament-like CARD architecture distinct from canonical CARD assemblies. Peptide- and LPS-induced CARD assembly require overlapping structural determinants, suggesting that structurally distinct ligands share common features in promoting higher-order CARD assembly. CBM7 also promotes oligomerization across inflammatory caspase CARDs and induces caspase activation, pyroptosis, and inflammatory responses in cells and mice. Together, these findings provide structural insight into ligand-induced inflammatory caspase CARD assembly and establish CBM7 as a chemically defined probe for dissecting the structural basis of inflammatory caspase activation.

## INTRODUCTION

Pyroptosis is a pro-inflammatory form of programmed cell death that plays a central role in host defense against infection and tissue damage^1,2^. Upon sensing pathogen-associated and damage-associated molecular patterns (PAMPs and DAMPs), cells initiate inflammatory signaling pathways that eliminate infected cells and amplify local immune responses^3–5^. During this process, specific pattern-recognition receptors (PRRs) assemble into multiprotein signaling complexes known as inflammasomes^3,4,6^. These complexes promote activation of inflammatory caspases, including caspase-1, caspase-4, caspase-5, and their murine homolog caspase-11 (CASP1, CASP4, CASP5, and CASP11), which mediate pyroptotic cell death^4,5^.

Canonical and non-canonical inflammatory caspases share high sequence similarity within their CARDs^7^. However, they are recruited and activated through distinct mechanisms^5,8^. In canonical inflammasomes, CASP1 serves as the main effector^4,6^. Upstream sensors and adaptor proteins, such as NOD-like receptors and ASC, provide CARD-containing scaffolds (inflammasomes) that recruit CASP1 through heterotypic CARD-CARD interactions^9,10^. Subsequent CARD-mediated clustering promotes CASP1 activation^11,12^. Activated CASP1 processes the pro-inflammatory cytokines IL-1β and IL-18 and cleaves the pore-forming protein gasdermin D (GSDMD), ultimately triggering pyroptotic cell death^4,13,14^.

In contrast, CASP4 and CASP5 in humans (and CASP11 in mice) mediate the non-canonical inflammasome pathway and do not require adaptor proteins for their initial activation^15^. Instead, their CARDs directly function as PRRs for cytosolic LPS, a major component of the outer membrane of gram-negative bacteria^16^. LPS consists of lipid A, a core oligosaccharide, and a highly variable O-antigen polysaccharide^17^. Lipid A is primarily responsible for non-canonical inflammasome activation^16,18^. LPS binding promotes CARD oligomerization and inflammatory caspase activation^15,16^. Activated non-canonical caspases cleave GSDMD, leading to pyroptosis and inflammatory responses^19,20^.

The oligomerization structures of CASP1^CARD^ and other canonical inflammasome CARD assemblies have been extensively studied and are known to adopt defined filament architectures^10,11^. In contrast, the higher-order assemblies formed by CARDs of non-canonical caspases remain poorly characterized.

LPS is currently the only ligand known to robustly induce CARD oligomerization of CASP4/5/11^16,21^. Several other lipids, including oxPAPC, cardiolipin, lipid IVa, and LPS-Rs, have also been reported to interact with CASP4^CARD^. However, none have been conclusively shown to induce comparable CARD oligomerization or activation, and some instead act as competitive inhibitors of LPS-mediated signaling^16,21–24^. Although biochemical and biophysical studies have identified potential LPS-responsive regions^16,18,24,25^, the structural organization of LPS-induced non-canonical CARD assemblies remains unresolved. Direct visualization of these assemblies has been challenging, likely because of the complex nature of LPS-protein interactions, including multivalent binding, ligand sharing between receptors, and the intrinsic tendency of LPS to form heterogeneous micelles in solution^21,24–26^. Alternative ligands capable of activating inflammatory caspase CARDs may therefore provide valuable tools for dissecting CARD oligomerization mechanisms independent of the structural complexity introduced by LPS.

SERPINB1 is a member of the serine protease inhibitor superfamily and belongs to the intracellular clade B serpins, being expressed in a wide range of cell types^27,28^. Beyond its classical role as a serine protease inhibitor^29^, we have previously shown that SERPINB1 suppresses inflammatory caspases, known as cysteine proteases^30^, through a non-enzymatic inhibitory mechanism^31^. A C-terminal CARD-binding motif (CBM) within SERPINB1 interacts with inflammatory caspase CARDs, preventing further CARD recruitment and oligomerization and thereby blocking caspase activation^31^. Deletion of a seven-amino-acid segment within this motif completely abolishes CARD binding^31^, indicating that this minimal sequence is critical for the interaction. These observations raise the possibility that a short SERPINB1-derived peptide could serve as a defined ligand for investigating CARD oligomerization mechanisms. However, whether the isolated SERPINB1-derived CBM can directly engage inflammatory caspase CARDs and influence CARD assembly remains unknown.

Here, we investigated whether a SERPINB1-derived peptide is sufficient to drive higher-order assembly of inflammatory caspase CARDs. We identified a seven-amino-acid peptide, CBM7, that directly binds the CASP4^CARD^ and induces its oligomerization *in vitro*. Using structural, biochemical, and functional approaches, we characterized peptide-induced CASP4^CARD^ assemblies and compared their interaction surfaces with those engaged by LPS. Notably, CBM7 induced oligomerization not only of CASP4^CARD^ but also of multiple inflammatory caspase CARDs, suggesting a shared mechanism of ligand-responsive CARD assembly. Consistent with these structural findings, cell-permeable CBM7 derivatives activated inflammatory caspases, triggered pyroptotic cell death, and elicited inflammatory responses in cells and mice. Together, these findings identify CBM7 as a defined molecular probe of inflammatory caspase CARD assembly and reveal general principles of ligand-induced CARD oligomerization that underlie non-canonical inflammasome activation.

## RESULTS

### A SERPINB1-derived peptide is sufficient to bind CASP4^CARD^ and drive higher-order assembly

Previous studies identified amino acids 358-364 of SERPINB1 as essential for its interaction with CARD of inflammatory caspases^31^ (Fig. 1a). To determine whether this minimal motif is sufficient for CARD recognition, we synthesized a seven-residue peptide corresponding to SERPINB1 residues 358-364 (358-FLFFIRH-364), hereafter termed CBM7 (Fig. 1a, b). A triple-phenylalanine-to-alanine mutant peptide (CBM7-M1) was generated and used throughout the study as a loss-of-function control (Fig. 1a).

While LPS is a stimulus for CASP4^CARD^, monomeric CASP4^CARD^ has been readily purified from both bacterial and non-bacterial expression systems^16,18,24–26,32^. For this reason, we expressed CASP4^CARD^ in *E. coli* as an N-terminal MBP fusion with a C-terminal SUMO tag to improve solubility. Size-exclusion chromatography (SEC) revealed both oligomeric and monomeric populations (**Supplementary Fig. 1a**). Because the oligomeric fraction was likely generated by endogenous bacterial LPS, only the monomeric fraction was purified and used for all subsequent analyses (**Supplementary Fig. 1b**).

We first compared whether CBM7 directly binds CASP4^CARD^ using biolayer interferometry (BLI). CBM7 bound CASP4^CARD^ with an apparent dissociation constant (K_D_) of 4.46 μM, whereas CBM7-M1 exhibited no detectable binding (Fig. 1c, d). This result indicates that the minimal SERPINB1-derived peptide is sufficient for direct recognition of CASP4^CARD^ and that the three phenylalanine residues are essential for this interaction.

CARD oligomerization is a critical step in inflammatory caspase activation and downstream pyroptotic signaling^3^. We therefore tested whether CBM7 binding is sufficient to promote CASP4^CARD^ assembly. GST-tagged CASP4^CARD^ purified from HEK293T cells was incubated with CBM7, the inactive CBM7-M1, or LPS as a reference ligand. CBM7 induced robust oligomerization of GST-CASP4^CARD^, whereas CBM7-M1 had no detectable effect (Fig. 1e). LPS was substantially more potent than CBM7, with 6 μM LPS producing a level of oligomerization comparable to that induced by approximately 250-400 μM of CBM7 based on native gel band migration patterns (Fig. 1e). In contrast CBM7-M1 failed to induce CASP4^CARD^ oligomerization even at 500 μM (Fig. 1e), consistent with its lack of detectable binding the BLI analysis data. Collectively, these results demonstrate that direct binding of the minimal SERPINB1-derived peptide is sufficient to drive CASP4^CARD^ oligomerization and that this activity critically depends on the three phenylalanine residues.

To enhance the ability of the peptide to induce CARD oligomerization, we next explored modifications of CBM7 (**Supplementary Fig. 1c, d**) and serendipitously found that conjugation to fluorescein (FITC) markedly increased its apparent affinity for CASP4^CARD^ (Fig. 1f). Consistent with this, FITC-CBM7, but not FITC-CBM7-M1, induced robust CASP4^CARD^ oligomerization at substantially lower peptide amounts than unconjugated CBM7, exhibiting more than tenfold greater potency (Fig. 1g). Peptide-induced oligomerization was further confirmed by size-exclusion chromatograph (SEC) analysis, which revealed the appearance of a new oligomeric species that was absent from the protein or peptide alone and resembled the oligomeric profile induced by LPS (Fig. 1h, **Supplementary Fig. 1e, f**). Together, these findings identify CBM7 as a minimal ligand capable of driving CASP4^CARD^ assembly and establish FITC-CBM7 as a potent tool to induce CARD oligomerization.

### Architecture of FITC-CBM7-induced CASP4^CARD^ assemblies

To gain structural insight into FITC-CBM7-induced CASP4^CARD^ oligomerization, we analyzed assembled complexes by negative-stain electron microscopy (nsEM). To improve protein yield and increase particle size for visualization, MBP and SUMO were fused to the N- and/or C-termini of CASP4^CARD^. Neither the MBP-CASP4^CARD^-SUMO construct nor FITC-CBM7 alone produced discernible structures by nsEM (Fig 2a). In contrast, addition of FITC-CBM7 to MBP-CASP4^CARD^-SUMO produced elongated rod-shaped assemblies (Fig. 2a). Similar assemblies were observed with constructs containing N- or C-terminal fusion partners, indicating that FITC-CBM7-induced oligomerization is largely independent of MBP or SUMO fusions (Fig. 2a). In the MBP fusion constructs, distinct peripheral densities corresponding to MBP were visible on the exterior of the assemblies regardless of whether MBP was fused to the N or C terminus (Fig 2a), suggesting that both termini of CASP4^CARD^ remain solvent exposed within the oligomer. Together, these findings indicate that FITC-CBM7 induces a robust assembly architecture that accommodates bulky terminal fusions without disrupting higher-order organization.

To obtain higher-resolution structural information, we next analyzed FITC-CBM7-induced CASP4^CARD^ assemblies by single-particle cryoEM. The overall morphology closely resembled the elongated filament-like assemblies observed by nsEM (Fig. 2a, **Supplementary Fig. 2a**), indicating that the same high-order architecture was preserved under cryogenic conditions. However, the particles exhibited substantial conformational flexibility, which hindered particle alignment and limited the achievable reconstruction resolution reconstruction (**Supplementary Fig. 2a**). As a result, the density map did not permit unambiguous localization of FITC-CBM7 within the filament.

Notably, this pronounced conformational flexibility appears to be a distinctive structural feature of CASP4^CARD^. In contrast to previously characterized CARD family filaments^3,33–37^, which generally adopt rigid, highly extended filament architectures (**Supplementary Fig. 2b**), CASP4^CARD^ assemblies exhibited marked structural plasticity. Consistent with the nsEM analysis, MBP-CASP4^CARD^ assemblies retained the flexible morphology, indicating that MBP fusion does not substantially alter filament-like assembly formation (Fig 2a, **Supplementary Figure 2a**).

To define the architecture of the filament-like assemblies, we performed extensive 2D classification analysis of the cryoEM data. Analysis of side and end-on views revealed a previously unrecognized two-layered architecture composed of distinct inner and outer layers (Fig. 2b–f). This structural feature was consistently observed in both the CASP4^CARD^-only construct and the MBP-CASP4^CARD^ construct (Fig. 2b, c), indicating that it is an intrinsic property of the assembly rather than an artifact of the fusion partner. Consistent with this unique architecture, the CASP4^CARD^ filament-like assemblies measured approximately 40 Å in diameter, nearly half the diameter of previously reported CARD filaments, including the ~80 Å CARD8^CARD^ filament (Fig. 2d, **Supplementary Fig. 2b**)^3,33–37^. Together, the compact diameter and distinctive two-layered organization demonstrate that CBM7-induced CASP4^CARD^ assemblies adopt an architecture fundamentally different from previously characterized CARD family filaments.

Among the resulting 2D class averages, a subset of top-view classes exhibited an apparent threefold symmetric arrangement (Fig. 2e). In these classes, the threefold organization was most prominent in the inner layer, whereas the outer layer appeared less well resolved. To explore a structural model consistent with these observations, we generated AlphaFold predictions using five CASP4^CARD^ molecules together with five CBM7 peptides. One predicted model exhibited a three-protomer arrangement in the end-on view that closely matched the overall diameter of the *ab initio* 3D reconstruction (Fig. 2f). Furthermore, this model enabled tentative assignment of predicted helical structural features corresponding to the double-layered density observed in the cryoEM map (Fig. 2f). Notably, both the cryoEM density and the AlphaFold model support a three-protomer organization, in contrast to the four-protomer architecture characteristic of previously reported CARD family filaments (**Supplementary Fig. 2b**). This alternative organization provides a plausible structural explanation of the unusually compact diameter of CASP4^CARD^ filament. Given that filament bending rigidity scales strongly with radial dimensions^38^, the reduced diameter, together with its distinct inter-protomer organization, may contribute to the pronounced structural flexibility of the CASP4^CARD^ assembly observed in the cryoEM images.

### CASP4^CARD^ residues 21-29 stabilize higher-order oligomer assembly

To further define the molecular interactions underlying CBM7-induced CASP4^CARD^ assembly, we performed hydrogen-deuterium exchange mass spectrometry (HDX-MS) using purified CASP4^CARD^-SUMO in the absence or presence of FITC-CBM7 or the inactive control peptide FITC-CBM7-M1. FITC-CBM7 induced broad reductions in deuterium uptake across multiple regions of CASP4^CARD^ (Fig. 3a, **Supplementary Fig. 3a**), whereas FITC-CBM7-M1 produced an HDX profile that closely resembled protein alone (**Supplementary Fig. 3b**), consistent with the requirement for the three phenylalanine residues in peptide-CARD interaction. Within this overall protection pattern, a prominent hotspot was centered on the 21-FLTGVLDNL-29 region, identified by a series of overlapping peptides that exhibited marked reductions in deuterium exchange upon FITC-CBM7 binding (Fig. 3a, b, **Supplementary Fig. 3c**).

To determine whether this protected region contributes to the proposed assembly architecture, we mapped residues 21-29 onto the AlphaFold-predicted model. This segment localized to the first α-helix (H1) of each protomer within the threefold symmetric core and occupied the protomer-protomer interface (Fig. 3c). In the predicted model, residues 21-29 contribute to inter-protomer contacts while also interacting with the second (H2) and third (H3) helices within the same protomer, suggesting a potential role in both oligomer assembly and intramolecular structural stabilization (Fig. 3c). To test this prediction experimentally, four hydrophobic residues (F21, L22, V25, and L26) within this CASP4^CARD^ 21-29 region were simultaneously substituted with alanine. Whereas the wild-type CASP4^CARD^ readily formed FITC-CBM7-induced oligomeric assemblies, the quadruple mutant failed to undergo oligomerization under the same conditions (Fig. 3d). These findings provide experimental support for the proposed structural model and identify residues 21-29 as a key determinant of CASP4^CARD^ oligomerization.

To determine whether the assembly defect resulted from impaired peptide binding, we next examined the predicted CBM7-CASP4^CARD^ binding interface. The AlphaFold model indicated that residues 21-29 are not positioned at the peptide-binding interface (**Supplementary Fig. 3d-f**). Consistent with this prediction, SPR analysis showed that the CASP4^CARD^ F21A/L22A/V25A/L26A mutant retained direct binding to FITC-CBM7, although its affinity (K_D_: 4.6 μM) was reduced by approximately fivefold compared with the wild type CASP4^CARD^ (K_D_: 0.9 μM) (Fig. 3e). These results indicate that disruption of residues 21-29 compromises oligomer assembly while retaining measurable peptide binding. Together, these findings support a two-step mechanism in which FITC-CBM7 initiates CASP4^CARD^ oligomerization, whereas residues 21-29 stabilize the assembled structure through coordinated inter- and intra-protomer interactions.

### CBM7 and LPS induce CASP4^CARD^ oligomerization through a shared structural framework

Following identification of residues 21-29 as an important determinant of oligomer stabilization, we next asked whether FITC-CBM7 and LPS promote CASP4^CARD^ assembly through a common structural mechanism. Although residues 21-29 represented one of the most strongly protected regions and were critical for oligomerization, HDX-MS also revealed ligand-induced reductions in deuterium exchange across multiple regions of the CARD (Fig. 3a, **Supplementary Fig. 3a-c**). Notably, a broadly similar protection pattern has also been reported for LPS-bound CASP4^CARD^, including pronounced protection near residues 21-29 together with widespread reductions in deuterium exchange throughout the CARD^24,25^. These similarities suggest that FITC-CBM7 recapitulates key structural features of LPS-induced CARD assembly, despite the distinct chemical nature of the two ligands.

We therefore compared peptide- and LPS-induced assemblies using complementary structural and biochemical approaches. To test whether the ligand-induced protection patterns identify residues important for oligomerization, we selected representative protected regions for mutational analysis. HDX-MS identified two major protected regions within CASP4^CARD^: an N-terminal hotspot centered on residues 21-29, and a broader, more moderately protected region spanning the C-terminal half of the domain, including residues 57-61 and 64-73 (**Supplementary Fig. 3c, 4a**). Guided by the protection profiles, we generated a series of mutants targeting the major protected cores and their neighboring regions. These included substitutions within the 21-29 hotspot (F21A/L22A/V25A/L26A and D27K), deletion of the adjacent N-terminal segment (Δ9-13), mutations within the C-terminal protected regions (D59K and K64A/R66A), and substitutions of hydrophobic residues located within or immediately downstream of the protected region (L72A/L73A and F76A/F77A).

Each mutant was then examined for its ability to undergo oligomerization in response to either FITC-CBM7 or LPS. Across all mutants tested, the two ligands produced remarkably similar oligomerization profiles, with most mutations exhibiting comparable effects under both conditions (Fig. 4a–c). Mutations in the N-terminal protected region had the strongest impact on oligomerization. The F21A/L22A/V25A/L26A mutant showed the most severe defects in oligomerization, followed by the Δ9-13 mutant (Fig. 4a–c). Although the hydrophobic mutants L72A/L73A and F76A/F77A at C terminus also reduced oligomerization, their effects were generally weaker than those of the N-terminal mutants (Fig. 4a–c). Notably, none of the charged-residue mutations caused a substantial loss of oligomerization (Fig. 4a–c). Together, these results indicate that FITC-CBM7- and LPS-induced CASP4^CARD^ oligomerization depend on a common set of structural determinants. In both cases, oligomerization depends most strongly on the N-terminal hydrophobic core, with more modest contributions from the C-terminal protected regions, whereas charged residues within these hotspots play comparatively limited roles.

Previous studies have suggested that the extreme C-terminal tail (residues 81-91) is dispensable for LPS recognition^26,39^. Likewise, FITC-CBM7 binding induced little change in deuterium exchange within residues 78-91, indicating that this region undergoes minimal structural rearrangement during ligand-induced assembly (**Supplementary Fig. 3c**). To define its functional contribution, we generated C-terminal truncation mutants. MBP-CASP4^CARD^(2-80) retained ligand-induced oligomerization comparable to that of full-length MBP-CASP4^CARD^(2-91) in response to both FITC-CBM7 and LPS (Fig. 4d–f). In contrast, MBP-CASP4^CARD^(2-75) exhibited little or no oligomerization activity under either condition (Fig. 4d–f). Consistent with these observations, SEC analysis revealed robust ligand-induced oligomerization of the full-length construct but not MBP-CASP4^CARD^(2-75) (Fig. 3g–i).

Together, these findings further support the conclusion that FITC-CBM7 recapitulates the structural requirements for LPS-induced CASP4^CARD^ assembly. Although the extreme C-terminal tail (residues 81-91) is dispensable, the adjacent 76-80 region is essential for productive oligomerization induced by both ligands. The limited changes in deuterium exchange across this region further suggest that it contributes primarily to maintaining the structural integrity required for assembly rather than undergoing major ligand-induced conformational rearrangements.

### CBM7 peptide broadly induces assembly across inflammatory caspase CARDs

Given the sequence similarity among inflammatory caspase CARDs (CASP1, 4, and 11)^40^ (Fig. 5a) and previous evidence that the SERPINB1 CBM interacts with multiple inflammatory caspases^31^, we next investigated whether CBM7 could promote assembly beyond CASP4^CARD^. To improve solubility and reduce spontaneous self-assembly, CASP1^CARD^ was expressed in bacteria as an MBP- and SUMO-tagged fusion construct based on a previously established design^10^. SEC analysis showed that MBP-CASP1^CARD^-SUMO was predominantly monomeric after purification (**Supplementary Fig. 4b**). However, following incubation at room temperature, BN-PAGE analysis revealed a detectable level of spontaneous oligomerization even with MBP and SUMO tags (Fig. 5b). Addition of CBM7 further enhanced CASP1^CARD^ oligomerization, and FITC-CBM7 promoted substantially stronger oligomerization than the unconjugated CBM7 peptide, mirroring its effects on CASP4^CARD^ (Fig. 5b).

To better visualize and compare peptide-induced assemblies, CASP1^CARD^-SUMO, CASP4^CARD^ and CASP11^CARD^ were analyzed under the same conditions. Because CASP1^CARD^ displayed a strong tendency toward spontaneous self-oligomerization, all proteins were maintained in the presence of 10% glycerol to minimize spontaneous assembly. Under these conditions, CASP1^CARD^ remained predominantly monomeric and showed no obvious higher-order assemblies by SEC or nsEM in the absence of peptide (Fig. 5c, d). In contrast, FITC-CBM7 induced clear oligomerization of CASP1^CARD^, and similar peptide-induced oligomers were also observed for CASP4 and CASP11 CARDs, even in the presence of 10% glycerol (Fig. 5d). Collectively, these observations indicate that CBM7 promotes oligomerization across multiple inflammatory caspase CARDs, suggesting that ligand-responsive CARD assembly may represent a conserved feature of this caspase family.

### TAT-CBM7 promotes inflammatory caspase assembly and activation in cells

We next examined whether peptide-induced CARD oligomerization observed *in vitro* could be recapitulated in cells. For cell assays, CBM7 was synthesized with an N-terminal TAT sequence to enhance solubility and facilitate cellular uptake^41^ (**Supplementary Fig. 5a**). HEK293T cells transiently expressing GFP-CASP4^CARD^ exhibited a marked increase in puncta following TAT-CBM7 treatment, whereas the inactive control peptide TAT-CBM7-M1 produced little effect (Fig. 6a, **Supplementary Fig. 5b**). Because transient overexpression can potentially increase background aggregation, we next examined cells stably expressing GFP-CASP4^CARD^. Under these conditions, TAT-CBM7 induced robust puncta formation comparable to that elicited by LPS, whereas TAT-CBM7-M1 produced almost no detectable puncta (Fig. 6b). These findings demonstrate that TAT-CBM7 promotes CASP4^CARD^ assembly in cells, consistent with its activity *in vitro*.

We then asked whether TAT-CBM7 similarly induces oligomerization of full-length inflammatory caspases. To prevent autocatalytic processing and cell death, catalytically inactive GFP-tagged CASP1^C285A^ and CASP4^C258A^ were transiently expressed in HEK293T cells. TAT-CBM7 strongly increased puncta formation for both full-length caspases, whereas TAT-CBM7-M1 showed minimal effects (Fig. 6c, d). Importantly, robust puncta formation was observed in the absence of FITC conjugation, demonstrating that the peptide itself is sufficient for intracellular CARD assembly.

We next investigated whether TAT-CBM7-induced CARD assembly in cells was functionally competent. Canonical inflammasome activation triggers CASP1 oligomerization and activation, followed by cleavage of IL-1β and GSDMD and secretion of mature IL-1β^3^. PMA-differentiated THP-1 macrophages were primed with LPS and then treated with TAT-CBM7 or TAT-CBM7-M1, with nigericin serving as a positive control^42,43^. Similar to nigericin, TAT-CBM7 induced cleavage of CASP1, IL-1β, and GSDMD and promoted secretion of mature IL-1β (Fig.6e, **Supplementary Fig. 5c**). Interestingly, CASP4 cleavage was also detected following TAT-CBM7 or nigericin treatment, whereas CASP5 processing remained minimal under the same conditions (Fig. 6e). In contrast, no activation of other CARD-containing or apoptosis-associated caspases, including CASP2, CASP3, or CASP9, was detected (Fig. 6e). Because canonical inflammasome activation requires a priming signal^3^, we also tested whether TAT-CBM7 affected LPS-dependent priming response. TAT-CBM7 did not alter LPS-induced TNF-α or IL-6 production (**Supplementary Fig.5d, e**), indicating that TAT-CBM7 acts downstream of the priming step. Finally, to examine non-canonical inflammasome signaling, THP-1 monocytes were treated with TAT-CBM7, with intracellular LPS delivery by electroporation used as a positive control. Both treatments induced GSDMD cleavage (Fig. 6f), consistent with activation of the CASP4-dependent non-canonical inflammasome pathway. Together, these data indicate that CBM7-induced CARD assembly is functionally competent and sufficient to drive inflammatory caspase activation and downstream pyroptotic signaling in cells.

### TAT-CBM7 triggers inflammatory caspase-dependent pyroptosis

Having established that TAT-CBM7 induces CARD assembly and inflammatory caspase activation in cells, we next examined whether this signaling is sufficient to trigger pyroptotic cell death. Treatment of monocytic cells with TAT-CBM7 induced rapid cell death, with an EC_50_ of approximately 10 μM and detectable activity within 60 minutes (Fig. 7a, **Supplementary Fig. 5f**). Live-cell imaging revealed rapid cell swelling following peptide treatment (**Supplementary Video**), a hallmark of pyroptosis^44^. Transmission Electron Microscopy (TEM) further showed characteristic features of pyroptotic cell death^45–47^, including plasma membrane rupture, cytoplasmic translucency, organelle swollen, and nuclear rupture at later time points. These features were not observed in TAT-treated cells (Fig. 7b, **Supplementary Fig. 5g**). To confirm that peptide-induced cell death depended on inflammatory caspase signaling, THP-1 cells lacking CASP1/4 or GSDMD were treated with TAT-CBM7. Both CASP1/4 knockout and GSDMD knockout cells were largely resistant to TAT-CBM7-induced cell death and failed to secret mature IL-1β (Fig. 7c, d). These findings indicate that TAT-CBM7 induces inflammatory caspase- and GSDMD-dependent pyroptosis.

We next examined the cell-type specificity and sequence requirements of peptide-induced cell death. Consistent with the known distribution of pyroptotic signaling pathways^44^, monocytic cell lines (THP-1 and U937) were the most sensitive to TAT-CBM7, whereas other blood-derived cell types exhibited substantially weaker responses (Fig. 7e). Structure-function analysis further showed that substitution of a single phenylalanine residue did not abolish activity, whereas replacement of two or three phenylalanine residues eliminated peptide-induced cytotoxicity (Fig. 7f). In contrast, mutation of the C-terminal arginine and histidine residues had little effect (Fig. 7f). Together, these findings identify the phenylalanine-rich hydrophobic motif as the principal determinant of peptide-induced pyroptosis, consistent with its essential role in CARD binding and oligomerization.

### TAT-CBM7 peptide activates inflammatory caspase signaling *ex vivo* and *in vivo*

We next examined whether TAT-CBM7 could induce inflammatory caspase signaling in primary cells and *in vivo*. Mouse bone marrow-derived macrophages (BMDMs) were isolated, differentiated, primed with LPS, and treated with peptides. CBM7 peptide induced robust secretion of mature IL-1β, whereas neither the mutant peptide nor BMDMs derived from *Casp1/11* KO mice produced comparable amounts of mature IL-1β (Fig. 8a). Consistent with our observations in THP-1 cells, TAT-CBM7 did not alter LPS-induced IL-6 production (**Supplementary Fig. 6a**), indicating that the peptide acts downstream of the priming step. These findings demonstrate that TAT-CBM7 activates inflammatory caspase signaling in primary macrophages.

To determine whether TAT-CBM7 also elicits inflammatory responses *in vivo*, TAT-CBM7 was injected intraperitoneally into C57BL/6 mice. Neither the WT nor mutant peptide resulted in significant body-weight loss, indicating no overt toxicity (**Supplementary Fig. 6b**). Flow cytometry analysis of peritoneal immune cells revealed that TAT-CBM7 induced rapid accumulation of neutrophils, monocytes, and CD11b^+^ macrophages, accompanied by a reduction in large peritoneal macrophages (LPMs) (Fig. 8b). These changes became evident within 2 h post-injection and became more pronounced by 5h (Fig.8c-f). In contrast, mice treated with TAT-CBM7-M1 displayed immune cell profiles that were largely indistinguishable from the PBS-treated controls (Fig. 8b, 8g-j). These results indicate that TAT-CBM7 is sufficient to trigger rapid inflammatory responses *in vivo*, consistent with activation of inflammatory caspase signaling.

## DISCUSSION

SERPINB1 is the only protein reported to interact with the CARDs of all inflammatory caspases and suppress their activation through a non-enzymatic mechanism that inhibits CARD self-oligomerization^31^. CBM7 represents the minimal SERPINB1 sequence sufficient for CARD binding. Unexpectedly, whereas full-length SERPINB1 suppresses CARD assembly, the isolated CBM7 peptide promoted higher-order CARD oligomerization and inflammatory caspase activation. One possible explanation is that the larger SERPINB1 protein sterically restricts the CARD-CARD interactions required for productive assembly, whereas the minimal peptide preserves ligand binding while permitting or facilitating these interactions. Although the precise basis for these opposing activities remains to be determined, our findings demonstrate that CBM7 functions as a distinct ligand rather than simply recapitulating the activity of its parent protein. This property enabled us to use CBM7 as a chemically defined molecular probe to dissect the structural and functional mechanisms of inflammatory caspase CARD assembly.

Many structurally characterized CARD family proteins assemble into rigid helical filaments through repetitive subunit interactions^9,34^. Given the elongated morphology of FITC-CBM7-induced CASP4^CARD^ assemblies, we initially anticipated that they would adopt a similar filament architecture. Unexpectedly, cryoEM revealed a compact assembly with conformational flexibility distinct from previously characterized CARD filaments. Because the current resolution does not permit definitive assignment of the repeating subunit organization, we refer to these structures conservatively as filament-like assemblies. Importantly, to our knowledge, this study provides the first structural visualization of oligomeric CASP4^CARD^ assemblies. The pronounced structural plasticity observed in these assemblies further raises the possibility that, unlike the relatively rigid CARD filaments described previously, non-canonical inflammatory caspase CARDs can assemble into more adaptable supramolecular structures that can accommodate chemically distinct ligands while preserving productive signaling. Although this hypothesis will require higher-resolution structural studies for validation, our findings suggest that ligand-induced non-canonical inflammatory caspase activation may utilize a supramolecular assembly mechanism fundamentally different from the canonical CARD filament paradigm. Consistent with this interpretation, multiple cryoEM reconstruction strategies failed to improve map quality despite reproducibly capturing the same overall architecture, supporting the view that the observed structural plasticity reflects an intrinsic property of the CASP4^CARD^ assembly rather than a technical limitation of image processing.

Comparison of the HDX-MS protection profiles generated by FITC-CBM7 and LPS provided important mechanistic insight into ligand-induced CASP4^CARD^ assembly. Despite their distinct chemical properties, both ligands induced remarkably similar protection patterns, particularly within Helix 1 and, to a slightly lesser extent, Helix 3. Together with previous HDX-MS studies^24,25^ of LPS-bound CASP4/11^CARD^, these findings suggest that chemically distinct ligands converge on a common structural mechanism for promoting non-canonical inflammatory caspase CARD assembly rather than engaging entirely different activation pathways. Because HDX protection can arise from direct ligand binding, oligomer-interface formation, intramolecular stabilization, or reduced conformational dynamics, HDX-MS alone cannot distinguish among these possibilities. However, integration of the HDX-MS data with AlphaFold modeling, binding measurements, and mutational analyses supports a two-step mechanism. In this model, ligand binding initiates CARD assembly, whereas the conserved hydrophobic surface centered on residues 21-29 functions primarily to stabilize higher-order oligomers rather than to mediate ligand recognition directly. Consistent with this interpretation, mutation of the Helix 1 hydrophobic patch markedly impaired oligomerization while preserving measurable peptide binding, demonstrating that ligand recognition and oligomer stabilization are mechanistically separable processes. More broadly, these findings suggest that non-canonical inflammatory caspase activation is governed not by the chemical identity of an individual ligand, but by its ability to engage a conserved assembly mechanism that drives productive CARD oligomerization.

Consistent with the HDX-MS analysis, mutational studies further demonstrated that disruption of hydrophobic residues within the protected regions similarly impaired both peptide- and LPS-induced oligomerization, whereas mutations of charged residues produced comparatively modest effects in both systems. Together, these findings reinforce the conclusion that chemically distinct ligands engage a conserved network of structural determinants to drive productive CASP4^CARD^ assembly, supporting a common mechanism of non-canonical inflammatory caspase activation despite fundamentally different molecular architectures of the ligands.

Beyond its mechanistic insights, CBM7 provides several practical advantages over LPS as an experimental tool. Unlike LPS, which is structurally heterogeneous and subject to batch-to-batch variability, CBM7 is a chemically defined ligand with uniform molecular composition and readily tunable sequence features. This enables systematic mutagenesis, truncation, and chemical modification approaches that are difficult to achieve with LPS. Importantly, CBM7 is not intended to serve as a physiological substitute for LPS. Rather, as a minimal CARD-binding ligand, it isolates the core process of ligand-induced CARD assembly from the additional structural and biological complexity associated with bacterial endotoxin. We therefore propose that CBM7 serves as a chemically tractable molecular probe for defining the fundamental principles of ligand-induced non-canonical inflammatory caspase CARD assembly and activation, while providing a versatile platform for future mechanistic and structural studies.

The use of CBM7 also uncovered a potentially conserved capacity for ligand-responsive assembly across inflammatory caspase CARDs. Although canonical and non-canonical inflammatory caspases share substantial sequence similarity, their physiological modes of activation are distinct. CASP1^CARD^ undergoes spontaneous self-oligomerization following inflammasome assembly but does not directly recognize LPS, whereas CASP4/11^CARD^ requires ligand-induced oligomerization initiated by direct LPS binding^10,33^. Consistent with these differences, non-canonical inflammatory caspases interact only weakly with canonical inflammasome adaptors such as ASC^48^, indicating that the two pathways are generally activated independently. Remarkably, however, CBM7 promoted oligomerization of both CASP1^CARD^ and CASP4/11^CARD^, suggesting that these proteins retain a shared intrinsic capacity for ligand-responsive CARD assembly despite their divergent physiological activation pathways. Whether peptide-induced CASP1^CARD^ assemblies adopt an architecture similar to CASP4/11^CARD^ or instead utilize the well-characterized self-association interfaces remains to be determined. Nevertheless, our findings raise the possibility that inflammatory caspase CARDs share an evolutionarily conserved assembly mechanism that can be engaged by chemically defined ligands independently of their native upstream signaling pathways. If confirmed by future structural studies, CBM7 would represent the first chemically defined ligand shown to directly promote assembly of both canonical and non-canonical inflammatory caspase CARDs, providing a unique tool for dissecting shared principles of inflammatory caspase activation.

FITC conjugation substantially enhanced the detection of CBM7-induced CASP4^CARD^ oligomerization in biochemical assays using purified protein, whereas unconjugated TAT-CBM7 was sufficient to induce inflammatory signaling in cultured cells and *in vivo*. Although the basis for this difference remains unclear, one explanation is that peptide-induced CASP4^CARD^ assemblies are relatively weak or dynamic under cell-free conditions and may partially dissociate during purification or biochemical analysis, making stabilization by FITC conjugation necessary for reliable detection. In contrast, the intracellular environment provides molecular crowding and additional cellular components that may facilitate or stabilize ligand-induced CARD assembly. Moreover, inflammatory signaling in cells may require only transient or lower-order CARD assemblies that fall below the detection threshold of biochemical assays. Thus, the threshold for initiating inflammatory signaling may be substantially lower than that required to detect stable higher-order oligomers *in vitro*. Although this model remains speculative, it is consistent with our biochemical and cellular observations and supports the idea that dynamic ligand-induced CARD interactions can be sufficient to initiate inflammatory caspase activation.

The ability of TAT-CBM7 to directly enter cells and activate inflammatory caspases also makes it a useful experimental tool for studying inflammatory caspase signaling. Unlike canonical inflammasome activation, which generally requires priming and secondary activation signals, or non-canonical inflammasome activation, which requires intracellular delivery of LPS, TAT-CBM7 induces inflammatory signaling following simple addition to the culture medium. This experimental simplicity facilitates investigation of CARD-dependent caspase activation without the need for ligand transfection or electroporation. At the same time, because TAT-CBM7 activates multiple inflammatory caspases, it should be applied cautiously in studies requiring selective activation of individual inflammasome pathways.

In summary, our study establishes CBM7 as a chemically defined molecular probe for dissecting ligand-induced inflammatory caspase CARD assembly. By integrating structural, biochemical, cellular, and *in vivo* analyses, we show that chemically distinct ligands can converge on conserved mechanisms of CARD oligomerization and inflammatory caspase activation. These findings further suggest that inflammatory caspase CARD assemblies possess an unexpected degree of structural plasticity while retaining common molecular principles that govern productive higher-order assembly. Although the precise architecture and physiological relevance of CBM7-induced assemblies remain to be fully defined, the experimental platform established here provides a foundation for future high-resolution structural studies and mechanistic investigations into the molecular basis of inflammatory caspase activation.

## METHODS

### Cloning, protein expression, and purification

For protein purification, His-MBP-CASP4^CARD^-SUMO, His-MBP-CASP4^CARD^ or His-MBP-SUMO was cloned into a modified pMAL vector, incorporating a TEV cleavage site between MBP and CARD, and a thrombin cleavage site between CARD and SUMO. GST-CASP4^CARD^ was cloned into the pEBG vector. For cellular studies, GFP, GFP-CASP4^CARD^, GFP-CASP1^C285A^, and GFP-CASP4^C258A^ were cloned into the pIRES vector. All plasmids were validated by Sanger sequencing. To purify CARDs from bacteria, constructs were transformed into BL21(DE3) cells. Protein expression was induced with 0.3 mM isopropyl β-d-1-thiogalactopyranoside (IPTG) when the culture reached an OD600 of 0.6, followed by overnight incubation at 18°C. Cell pellets were collected and lysed by sonication (5 s on, 10 s off, 2 min total at 30% power, on ice) in lysis buffer (20 mM Tris-HCl, pH 7.5, 150 mM NaCl, 5 mM β-mercaptoethanol, and a protease inhibitor cocktail). The lysate was clarified by centrifugation and filtration (0.22 μm) and applied to cobalt resin (Sigma) using gravity flow. The resin was washed with 5 column volumes (CV) of lysis buffer and eluted with 5 CV of elution buffer (20 mM Tris-HCl, pH 7.5, 150 mM NaCl, 250 mM imidazole, 5 mM β-mercaptoethanol). Protein concentrations in the elution fractions were estimated using a Nanodrop spectrophotometer. Elutions were purified by size-exclusion chromatography on a Superdex 200 column (Cytiva) using SEC buffer (20 mM Tris-HCl, pH 7.5, 150 mM NaCl). The final protein concentration was again estimated using Nanodrop. To remove SUMO, purified fusion protein was co-incubated with thrombin (Sigma) at a ratio of 2 units/mg with 10 mM CaCl_2_ at room temperature for 2 h, followed by His purification again to remove SUMO. To remove MBP, purified fusion protein was mixed with TEV at a ratio of 1:20 (w/w) with 1 mM DTT at 4°C overnight. The mixture was passed through the Cobalt resin and flow-through was collected. CASP1^CARD^ and CASP11^CARD^ were cloned into the same vector and obtained with the same protocol. 10% glycerol was added to all the buffers during CASP1^CARD^ purification to prevent self-oligomerization post adaptor removal. GST-fused CARD was purified from HEK293T cells. Transfection was performed with the relevant plasmids, and cells were incubated for 48 hours post-transfection. The harvested cell pellets were processed using the same purification protocol described above.

### Oligomerization analyses

When hydrophobic peptides were used, the reaction contained 4% DMSO (final concentration). LPS used for oligomerization assays are LPS, *E. Coli* O111:B4 (Sigma), Rd-LPS, *E. coli* F583 (Sigma), and Re-LPS, *S. enterica* serotype minnesota Re 595 (Sigma). Molarity estimates for smooth LPS from *E. Coli* O111:B4 were based on previously reported average molecular weights^49,50^. To facilitate more accurate estimation of LPS molarity, rough LPS were used in the subsequent experiments. Purified proteins were incubated with stimuli for 1 hour at room temperature. For bacterial purified proteins, oligomers were separated by BN-PAGE (Invitrogen) or by size-exclusion chromatography using a Superdex 200 column (Cytiva). Oligomers eluting away from the monomer peak were collected and concentrated for TEM or cryoEM studies. GST-CASP4^CARD^ Oligomers from HEK293T were separated by home-made pore-limit native PAGE as described before^16^.

### Negative-stain TEM

Protein samples (A280 = 0.05) were applied to Formvar/carbon-coated copper grids (Electron Microscopy Sciences) for 1 minute, followed by staining with 0.1% uranyl formate for an additional 1 minute. The grids were air-dried and imaged at 80 keV using an FEI Tecnai G2 Spirit BioTWIN microscope at the Lerner Research Institute Imaging Core.

### Grid preparation and cryoEM data collection

For cryoEM grid sample preparation, synthetic FITC-CBM7-bound purified CASP4^CARD^ or MBP-CASP4^CARD^ was diluted to 1 mg/ml in the final SEC buffer (phosphate buffer saline) immediately before grid preparation. C-flat holey carbon grids (R1.2/1.3, 300 mesh Cu) were glow-discharged for 30 s using a plasma cleaner (PELCO easiGlow, Ted Pella). A 3 μl sample was applied to freshly glow-discharged grids under 277K and 100% humidity conditions using a Vitrobot Mark IV (Thermo Fisher Scientific). Excess solution was removed by blotting with filter paper under blot force 0 and blot time 4-7s. The grids were then rapidly plunge-frozen into liquid ethane cooled by liquid nitrogen. Frozen grids were stored in liquid nitrogen until cryoEM grid screening and data collection.

CryoEM data were collected at the Cleveland Center for Membrane and Structural Biology (CCMSB) using a 200 kV Glacios cryo-transmission electron microscope (Thermo Fisher Scientific) equipped with an X-FEG electron source, Selectris energy filter, and Falcon IV direct electron detector. Grid screening, automated data acquisition, and remote monitoring were performed using the EPU software package. Movies were acquired in Falcon IV EER mode. For each exposure, a total electron dose of 40 e^−^/Å^2^ was fractionated across multiple frames. Defocus values were imposed over a range of −0.6 to −1.6 μm. Collected EER movie stacks were processed using cryoSPARC^51^ v4.7.1 for motion correction, dose-weighting, CTF estimation, particle picking, and downstream image processing.

### HDX-MS

HDX sample handling, mixing, and labeling were performed using an automated HDX system (Trajan Scientific and Medical, Morrisville, NC). The CASP4^CARD^-SUMO fusion protein (13.64 μM) was prepared in 20 mM Tris-HCl (pH 7.5) containing 150 mM NaCl and incubated either alone or in the presence of FITC-CBM7 or FITC-CBM7-M1. Protein-peptide complexes were generated by mixing protein and peptide to achieve a final peptide concentration of 108.8 μM, corresponding to an approximate protein-to-peptide molar ratio of 1:8. Samples were equilibrated at 0 °C for at least 10 min prior to HDX analysis.

Deuterium exchange reactions were initiated by mixing 5 μL of protein solution with a deuterated buffer consisting of 10 mM Tris prepared in D_2_O (pH read 7.5). For the undeuterated control (0 s time point), an identical buffer prepared in H_2_O was used. Exchange reactions were carried out at 25 °C for 0, 60, 180, and 600 s.

Exchange reactions were quenched by transferring 45 μL of the reaction mixture into 45 μL of prechilled quench buffer maintained at 0 °C and containing 2 M guanidine hydrochloride and 0.8% formic acid. This rapid reduction in pH and temperature minimized further deuterium exchange. Subsequently, 80 μL of the quenched sample was injected into the online digestion system for proteolytic processing.

Online digestion was performed using a Waters Enzymate BEH Pepsin column (2.1 × 30 mm) operated at a loading flow rate of 100 μL/min. Peptides were trapped on an ACQUITY UPLC Peptide BEH C18 VanGuard pre-column and separated on an ACQUITY UPLC BEH C18 analytical column (130 Å, 1.7 μm, 1 × 100 mm). Chromatographic separation was carried out at 40 μL/min using solvent A (water containing 0.1% formic acid) and solvent B (acetonitrile containing 0.1% formic acid). The gradient program was as follows: 5% B from 0-7 min, increased to 40% B at 7 min, ramped to 80% B at 8 min and held until 10 min, followed by re-equilibration at 5% B from 12-15 min.

Fully deuterated controls were prepared to estimate back-exchange and determine maximum deuterium incorporation. Briefly, 20 μL of CASP4^CARD^-SUMO (20.7 μM) was dried using a SpeedVac concentrator and reconstituted in 30 μL of 10 mM ammonium bicarbonate containing 5 M guanidine hydrochloride prepared in D_2_O. Samples were incubated at 45 °C for 2 h prior to analysis.

Separated peptides were analyzed using a Waters SELECT SERIES Cyclic IMS instrument (Waters Corporation, Milford, MA), a quadrupole time-of-flight (QTOF) mass spectrometer equipped with cyclic ion mobility separation. Peptides were ionized by electrospray ionization in positive ion mode and analyzed in V-mode using HDMSE acquisition. HDMSE is a data-independent acquisition method that integrates ion mobility separation with alternating low- and elevated-collision energy scans to generate multidimensional mass spectrometric data from complex peptide mixtures.

Leucine enkephalin [(M+2H)^2+^, m/z 556.2766)] was used as the lock-mass compound for post-acquisition mass correction. Spectra were acquired over an m/z range of 50-2000 using a capillary voltage of 3.0 kV, cone voltage of 40 V, and transfer collision energy ramped from 15 to 50 V. One-pass cyclic ion mobility (cIM) separation was performed using a sequence consisting of 10 ms ion injection, 2 ms ion mobility separation, and 34 ms ejection/acquisition.

### HDX-MS Data Analysis

Peptide mapping was performed using nondeuterated HDMSE datasets processed in ProteinLynx Global Server (PLGS) version 3.0.2 (Waters Corporation). Low- and elevated-energy count thresholds were set to 2500 and 500 counts, respectively. The digestion reagent parameter was set to nonspecific, and peptide identifications were filtered at a 1% false discovery rate. Peak lists were searched against the target protein sequence database.

PLGS search results and all raw mass spectrometry data files were subsequently imported into HDExaminer version 3.4.2 (Trajan Scientific and Medical) for deuterium uptake analysis. Automated peptide assignments were manually inspected and curated based on isotopic envelope quality, retention time consistency, drift time consistency, and overall signal quality. Deuterium uptake values were calculated for each peptide at all labeling time points and used for subsequent statistical analysis using HDXBoxeR^52^.

### Biolayer interferometry

BLI was performed using an Octet R2 system (Sartorius) with streptavidin (SSA) biosensors. Biotinylated CBM7 peptides were immobilized onto SSA sensors. Purified CASP4^CARD^ protein was applied at increasing concentrations, and binding was monitored during association and dissociation phases. Reference sensors without peptide coating were used to correct for non-specific binding. Steady-state analysis was used to determine apparent binding affinities in the Octet analysis software.

### SPR

SPR experiments were performed on Biacore S200 using CM5 sensor chips, modified from the described procedure^16^. Purified CASP4^CARD^ proteins were immobilized on the chip surface at a concentration of 10 μg/ml to a final coupling level of approximately 180 response units (RU), using standard amine-coupling chemistry. An uncoated flow cell was used as a reference channel. SPR measurements were carried out in HEPES buffer (pH 7.5) containing 150 mM NaCl and 2% (v/v) DMSO. FITC-CBM7 peptide was injected as the analyte with an association time of 5 min followed by a dissociation time of 5 min. Single injections were performed for each concentration to prevent precipitations. Regeneration of the sensor surface was achieved by injection of 20 mM NaOH for 15 s. Data is analyzed by TraceDrawer software, using the 1:1 binding model.

### Cell culture and transfection

All cells were cultured at 37°C in a humidified incubator with 5% CO_2_. HEK293T cells (ATCC) were maintained in DMEM supplemented with 10% fetal bovine serum (FBS; Gibco) and 1% penicillin-streptomycin (Pen/Strep; Gibco). U937 and THP-1 cells (both from ATCC) were cultured in RPMI medium supplemented with 10% FBS, 1% Pen/Strep, and 55 μM β-mercaptoethanol. Transfections were carried out using Lipofectamine 3000, following the manufacturer’s protocol.

### Pyroptosis activation and cell death assays

All peptides were synthesized by Biomatik or Genscript. U937 or THP-1 cells were incubated with the peptides in the presence of Sytox-Green or Propidium Iodide, and fluorescence intensity was measured using a Varioskan LUX microplate reader (Thermo Fisher Scientific) for up to 2 hours. Cell viability was assessed at the experiment’s endpoint by adding Cell Titer-Glo reagent and measuring luminescence. For LPS transfection, a total of 1 μg of LPS was mixed with 5×10^5^ cells and electroporated using the Neon system (Invitrogen). Transfected cells were then incubated in complete media without antibiotics for 0.5-2 hours. To induce pyroptosis in macrophages, U937 and THP-1 cells were differentiated with PMA for 1 day, followed by a 2-day rest in fresh media. The cells were then primed with LPS for 3-4 hours before treatment with peptides or nigericin for 1 hour in serum-free media. Supernatants were analyzed for IL-1β or IL-6 using ELISA kits (BD), following the manufacturer’s protocol.

### Immunoblots

Cell lysates or supernatants were mixed with SDS sample buffer, separated by SDS–PAGE, and transferred to PVDF membranes for immunoblot analysis. Membranes were blocked in milk and incubated with primary antibodies overnight, followed by HRP-conjugated secondary antibodies. The following antibodies were used: Anti-mouse-HRP (CST, #7076); Anti-rabbit-HRP (CST, #7074); Anti-GSDMD (CST, #97558); Anti-GSDME (Abcam, #ab215191); Anti-Pro-IL-1β (CST, #12242); Anti-IL-1β, p17 (CST, #83186); Anti-Caspase-1 (AdipoGen, #AG-20B-0048); Anti-Caspase-4 (Santa Cruz, #sc-56056), Anti-Caspase-5 (CST, #4429), Anti-Caspase-2 (BD, #611022), Anti-Caspase-3 (CST, #9665), Anti-Caspase-9 (CST, #9502). Images were acquired using a Bio-Rad ChemiDoc imaging system.

### Immunofluorescence imaging

For transient expression, 293T cells were transfected with plasmid DNA using PEI, then replated the following day. Stable 293T cell lines were directly plated on coverslips in 12-well plates. Cells were treated with 50 μM TAT-CBM7 or TAT-CBM7-M1 for 2 h. For LPS controls, 293T cells were transfected with 1 μg LPS using Lipofectamine 2000. After 2 hours, cells were washed with PBS, fixed with 4% paraformaldehyde, and stained with Hoechst. Samples were mounted and imaged on a Nikon Eclipse C1 confocal, Zeiss LSM510 confocal, or Keyence BZ-X710 microscope.

### BMDM isolation

To generate bone marrow-derived macrophages (BMDMs), bone marrow was flushed from the femurs and tibias, and red blood cells were subsequently removed. The remaining cells were then suspended in DMEM containing 20% L929 cell-conditioned medium as a source of M-CSF (prepared in-house), 10% FBS, 2 mM L-glutamine, 1 mM sodium pyruvate (Gibco), and 1% Pen/Strep. Cells were seeded onto 10 cm non-treated culture dishes at 1.5E7 cells/plate. On day 3 or 4, the cultures were refreshed with media containing 10% M-CSF. After a 7-day differentiation period, the BMDMs were collected using cold DPBS (Gibco) and plated at 3E5 cells/well in 24-well plates for downstream experiments.

### Peritoneal immune cell isolation and flow cytometry

Young female C57BL/6J mice (8-12 weeks old) were used for *in vivo* assays. Mice were injected intraperitoneally (i.p.) with either peptide or PBS for 2-5 hours. Peptides were administered at 20 mg/kg for the time-course assay and at 10 mg/kg for the WT versus M1 peptide comparison. Immune cells from the peritoneal cavity were collected by flushing twice with 7 mL of FACS buffer (PBS with 1% FBS). The cells were pre-incubated with Fc and monocyte blockers (Biolegend), then stained with the following antibodies: anti-Ly6G-APC (BD, #560599), anti-CD11b-eFluor450 (eBioscience, #48-0112-80), anti-CD19-BV605 (Biolegend, #115539), anti-F4/80-BV785 (Biolegend, #123141), anti-Ly6C-BV650 (Biolegend, #128049), and live/dead fixable blue/red (Invitrogen) in Brilliant Buffer (BD). After staining, the cells were washed and fixed with 4% PFA. Flow cytometry was performed using a Sony ID7000 at the Cleveland Clinic Research Flow Cytometry Core, and data were analyzed with FlowJo software. Cell gating was performed as described^53^. All animal experiments were approved by the IACUC of Cleveland Clinic and were performed in accordance with the NIH Guide for the Care and Use of Laboratory Animals and institutional guidelines.

### Statistical analyses

Unless otherwise stated, the statistical analyses were done with Prism 10. All experiments were performed with at least two to three independent biological replicates, as indicated by the data points presented on each graph. All statistical analyses were performed with two-tailed tests. Statistical significance was defined as: ns, not significant; *, P < 0.05; **, P < 0.01; ***, P < 0.001; and ****, P < 0.0001.

## Supplementary Material

This is a list of supplementary files associated with this preprint. Click to download.

- CBM7Figuressuppv64.pdf
- compressedSupplementaryVideo1.mov
- RS.pdf

## Figures and Tables

**Figure 1.**
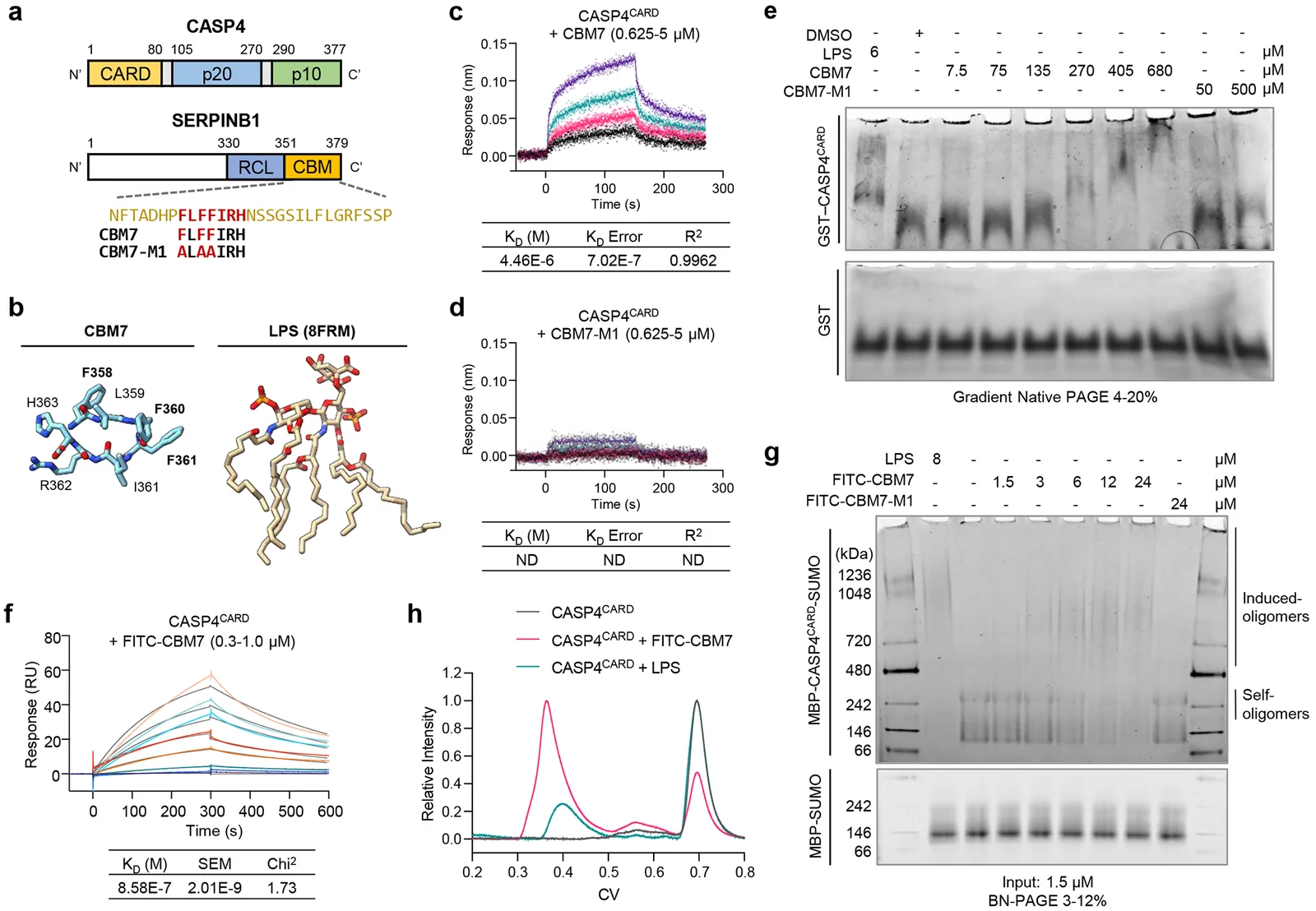
CBM7 peptide binds CASP4^CARD^ and initiates CARD oligomerization. **a**. Sequence information of SERPINB1-derived CBM7 peptide and the mutant variant. **b.** Structure information of CBM7 and *E. coli* LPS (PDB: 8FRM, containing lipid A and core oligosaccharides without the O-antigen polysaccharide). **c-d**. BLI analysis of CBM7 or CBM7-M1 binding to purified CASP4^CARD^. The concentrations used were 0.625, 1.25, 2.5, and 5 μM. Apparent equilibrium dissociation constant (K_D_) were obtained using steady-state analysis. **e**. Pore-limit Native Polyacrylamide Gel Electrophoresis analysis of CASP4^CARD^ in the presence of indicated concentration of LPS, CBM7, or CBM7-M1. **f**. SPR analysis of FITC-CBM7 binding to immobilized CASP4^CARD^. Peptide concentrations used were 0.3, 0.4, 0.5, 0.6, 0.8, 0.9, and 1.0 μM. Mean K_D_ values ± SEM are shown. **g**. Blue Native PAGE (BN-PAGE) analysis of MBP-CASP4^CARD^-SUMO oligomerization in the presence of LPS, FITC-CBM7, or FITC-CBM7-M1. **h**. SEC profiles of CASP4^CARD^ incubated with the indicated oligomerization inducers. Peak intensities were normalized for comparison.

**Figure 2.**
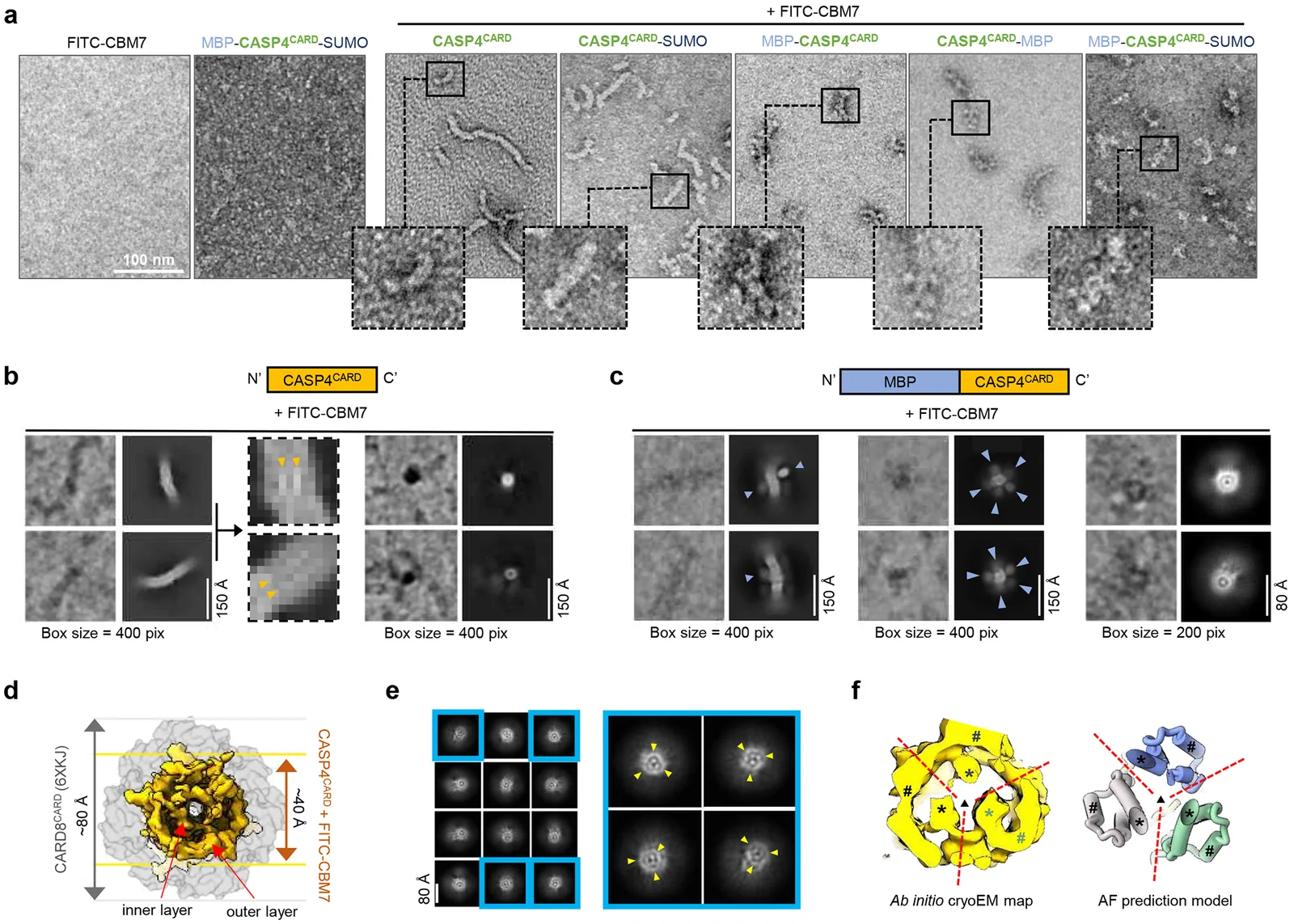
FITC-CBM7 drives filament-like assembly of CASP4^CARD^. **a**. Negative-staining electron microscopy analysis of CASP4^CARD^ constructs in the presence or absence of FITC-CBM7. **b**. Representatives of extracted particles and corresponding 2D class averages of filament-like assemblies from FITC-CBM7-induced and CASP4^CARD^-only samples. **c**. Representative extracted particles and 2D class averages of MBP-CASP4^CARD^ filament-like assemblies. **d**. *Ab initio* 3D model generated from top-view particles of CASP4^CARD^-only assemblies and structural superimposition with the previously reported CARD8^CARD^ filament-like assembly^35^. **e**. Representative 2D class averages of oligomers. Classes outlined in blue are enlarged in the right panel. Yellow arrowheads indicate the significant densities. **f**. Comparison between the *ab initio* cryoEM reconstruction and the AlphaFold prediction model. * marks the inner layer and # marks the outer layer.

**Figure 3.**
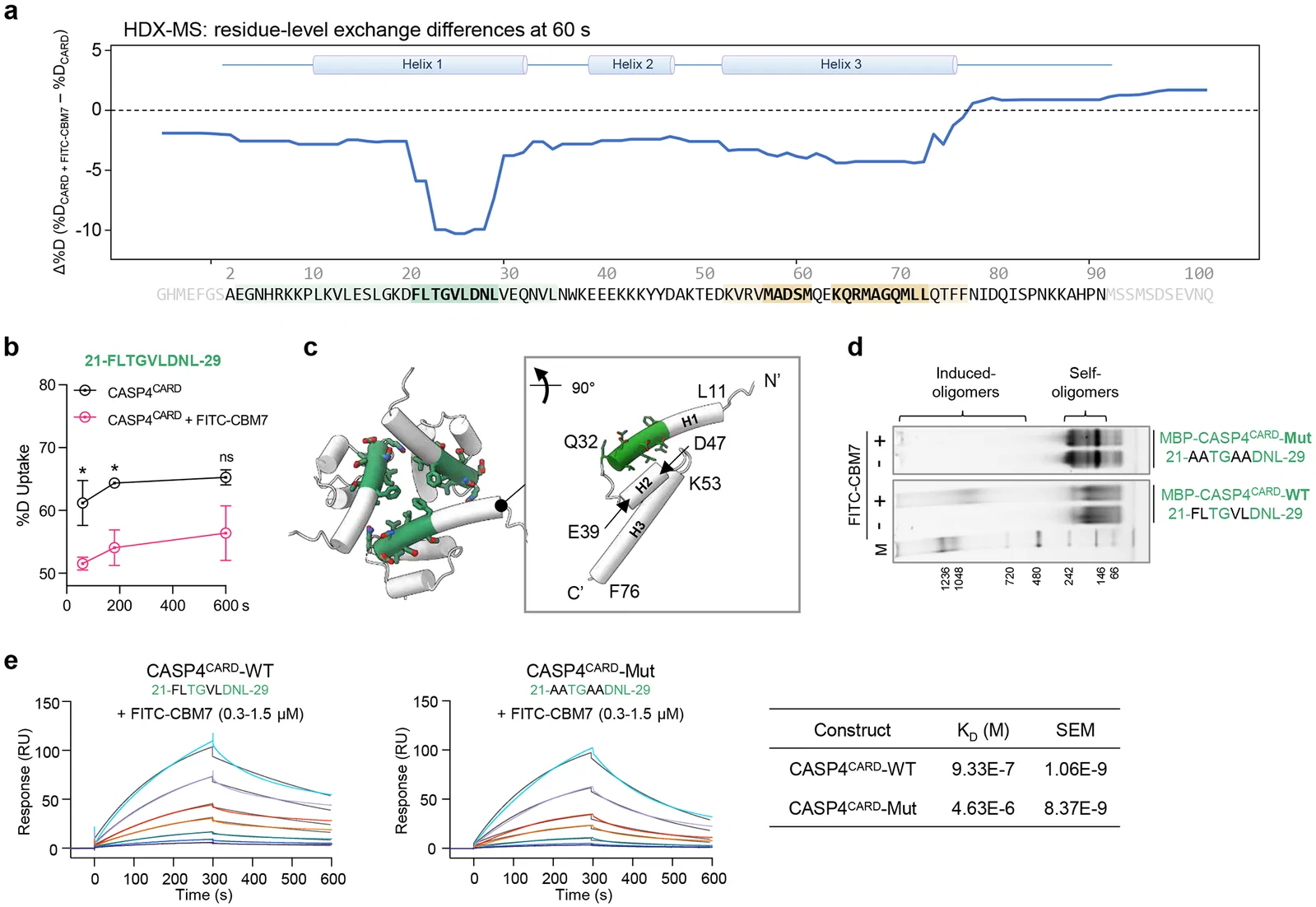
CASP4^CARD^ residues 21-29 stabilize higher-order oligomer assembly. **a**. Residue-level deuterium uptake differences between FITC-CBM7-bound and apo CASP4^CARD^ at 60 s exchange. Regions showing highly reduced deuterium exchange are highlighted. Grey labeled residues are N- and C-terminal linkers. The Helix information from AlphaFold prediction (same as Fig. 2h) is shown. **b**. HDX-MS analysis of CASP4^CARD^ in the absence or presence of FITC-CBM7. Deuterium uptake (%) of the peptide spanning residues 21-29 (FLTGVLDNL) was measured at the indicated labeling times. Data are presented as mean ± S.D. Statistical significance is indicated. **c**. AlphaFold prediction model of CASP4^CARD^. Residues 21-29 (FLTGVLDNL) identified in the HDX-MS analysis are highlighted in green. The right panel shows a magnified view. **d**. BN-PAGE analysis of induced oligomers of wild-type and mutant MBP-CASP4^CARD^ constructs in the presence or absence of FITC-CBM7. Molecular weight markers (kDa) are indicated. **e**. Representative SPR sensorgrams of FITC-CBM7 binding to wild-type CASP4^CARD^ (left) and the F21A/L22A/V25A/L26A mutant (right). Peptide concentrations used were 0.3, 0.45, 0.5, 0.6, 0.8, 1.0, and 1.5 μM. Mean K_D_ values ± SEM are shown.

**Figure 4.**
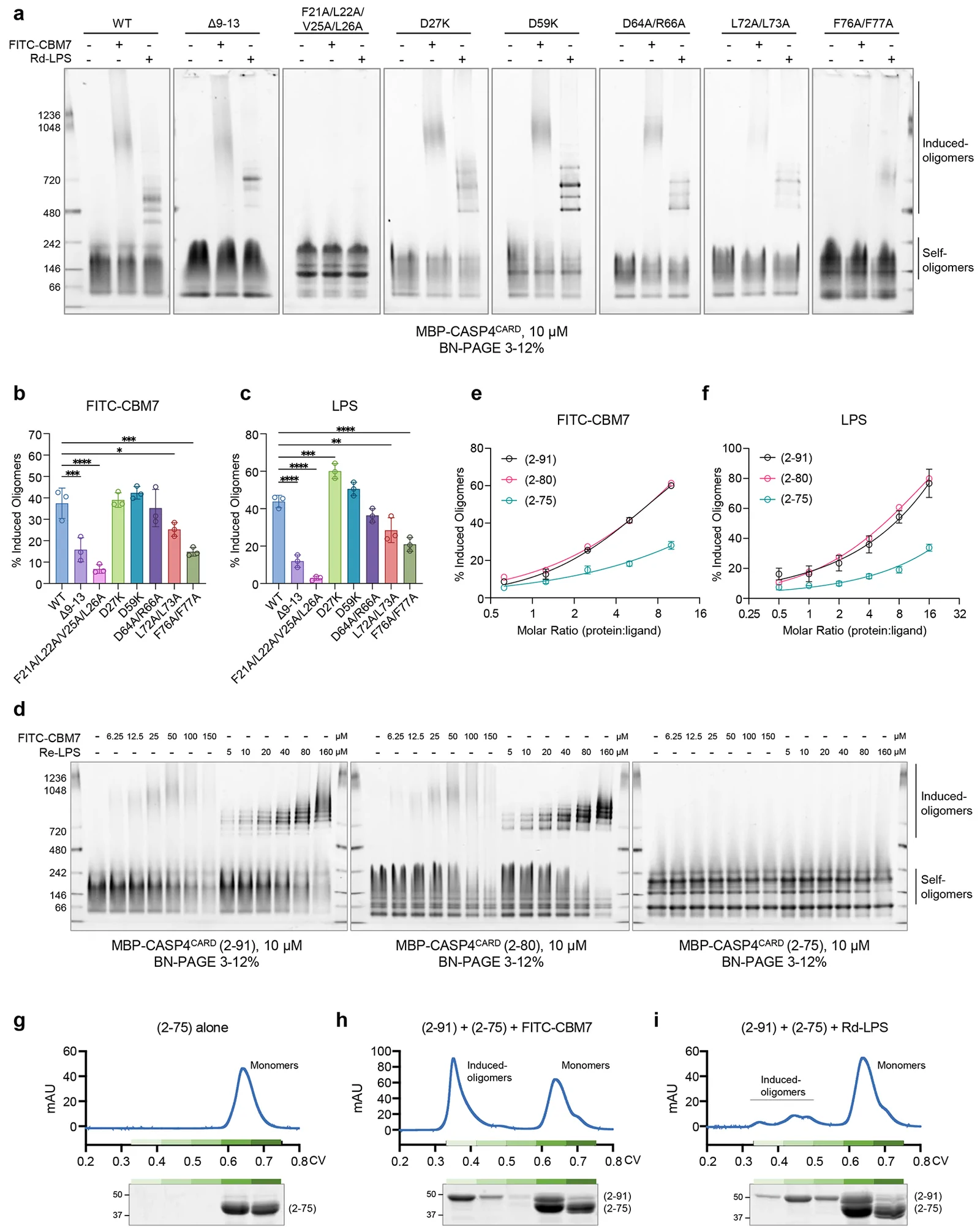
FITC-CBM7 and LPS induce CASP4^CARD^ oligomerization via a shared structural mechanism. **a-c.** BN-PAGE analysis of WT or mutant MBP-CASP4^CARD^ oligomerization induced by 50 μM CBM7 or 40 μM *E. coli* Rd-LPS. Induced oligomer fractions were quantified, and statistical significance was determined by one-way ANOVA. The p-values were only labeled for those showing significance. **d-f.** BN-PAGE quantification of WT or C-terminal truncation mutants of MBP-CASP4^CARD^ in the presence of the indicated concentrations of FITC-CBM7 or rough LPS. Induced oligomer fractions were quantified as described above. **g-i.** SEC analysis of MBP-CASP4^CARD^ (2-91) and/or (2-75) alone or following incubation with FITC-CBM7 or *E. coli* Rd-LPS. Fractions corresponding to major SEC peaks were grouped into five fractions (indicated by colored bars) and analyzed by SDS-PAGE.

**Figure 5.**
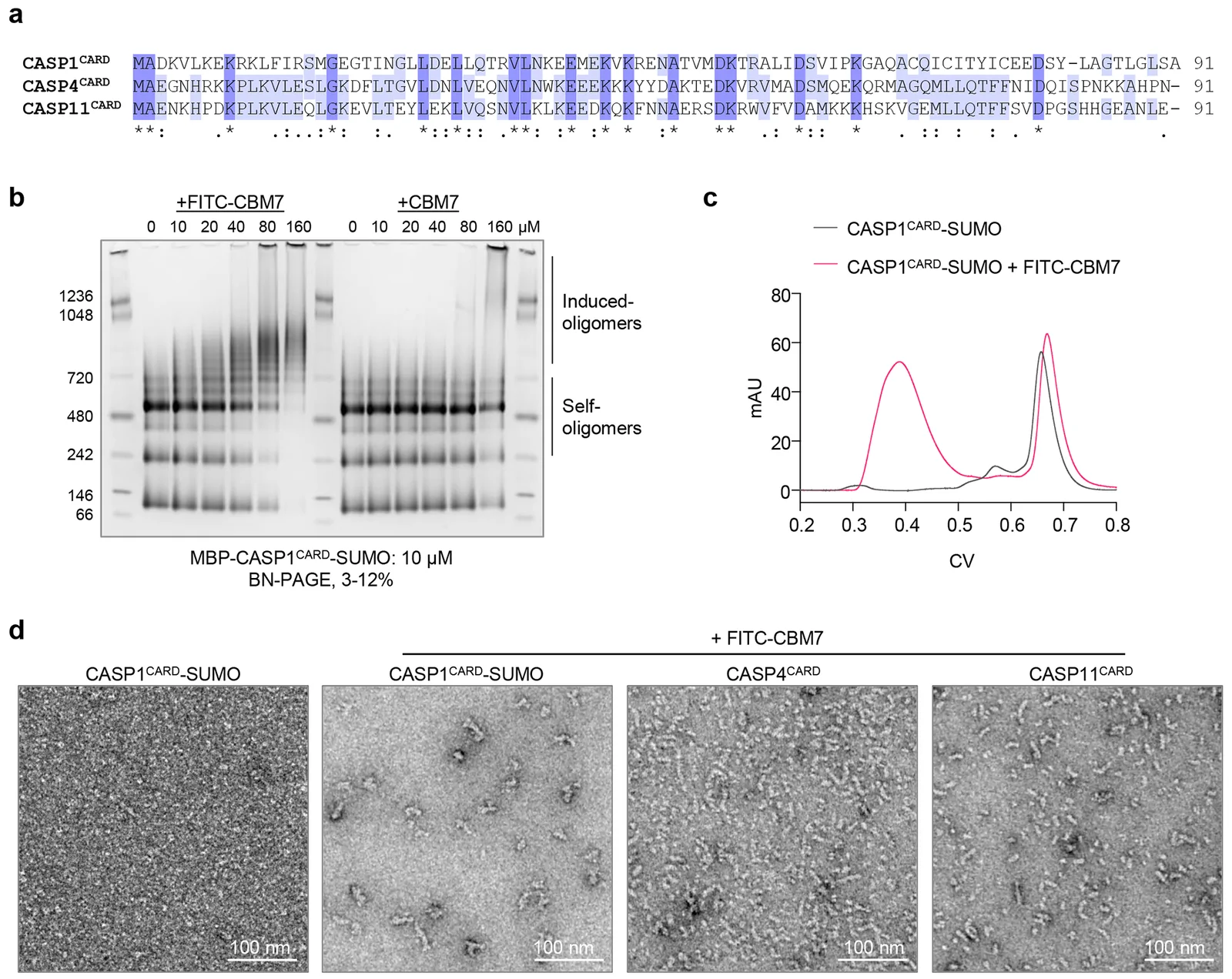
CBM7 peptide broadly induces assembly across inflammatory caspase CARDs. **a.** Sequence alignment of CARDs from CASP1, CASP4, and CASP11 by Uniprot Alignment tool. Identical amino acids were highlighted. (*) = identical; (:) = Conserved substitutions; (.) = Semi-conserved substitutions. **b**. BN-PAGE analysis of MBP-CASP1^CARD^-SUMO oligomerization induced by CBM7 or FITC-CBM7. **c**. SEC analysis of CASP1^CARD^-SUMO in the presence or absence of FITC-CBM7. **d**. Negative-stain EM images of CASP1^CARD^-SUMO alone, or CASP1, CASP4, or CASP11 CARDs in the presence of FITC-CBM7.

**Figure 6.**
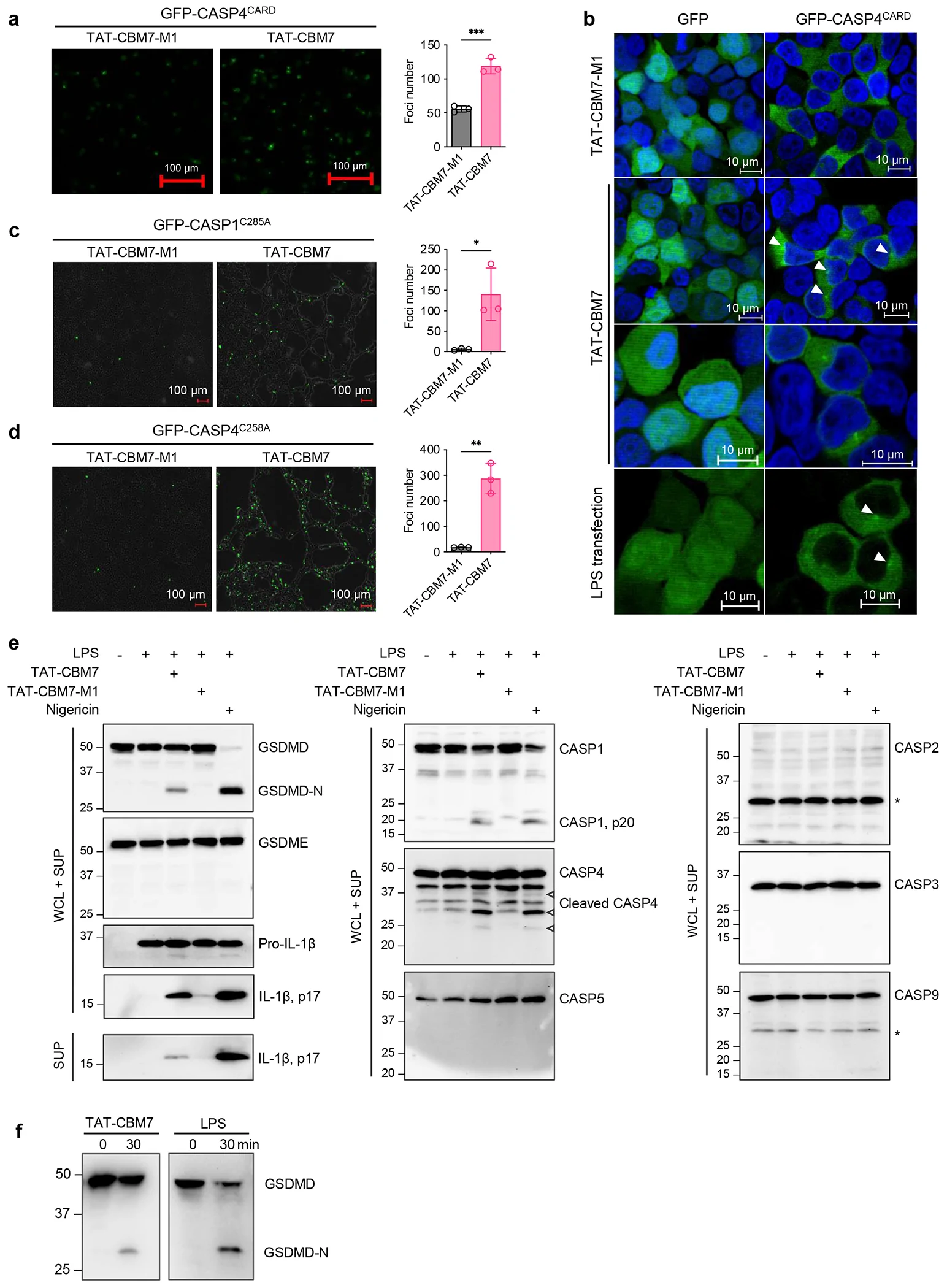
TAT-CBM7 induces CARD oligomerization and caspase activation in cells. **a**. GFP-CASP4^CARD^ puncta formation in HEK293T cells treated with TAT-CBM7 or TAT-CBM7-M1. Cells were analyzed by confocal microscopy 2 h after peptide treatment. Quantification was performed from three fields of view. A student t-test was performed for statistics. **b**. Puncta formation in HEK293T cells stably expressing GFP or GFP-CASP4^CARD^ following treatment with TAT-CBM7, TAT-CBM7-M1, or LPS transfection. Nuclei were stained with Hoechst. **c-d**. Puncta formation of overexpressed full-length CASP1^C285A^ or CASP4^C258A^ in HEK293T cells treated with TAT-CBM7 or TAT-CBM7-M1. Quantification was performed from three fields of view. A student t-test was performed for statistics. **e**. Immunoblot analysis of inflammasome activation in PMA-differentiated THP-1 macrophages treated with TAT-CBM7, TAT-CBM7-M1, or nigericin following LPS priming. Whole-cell lysates (WCL) and supernatants (SUP) were analyzed. **f**. Immunoblot analysis of GSDMD cleavage in THP-1 monocytes treated with TAT-CBM7 or electroporated with LPS.

**Figure 7.**
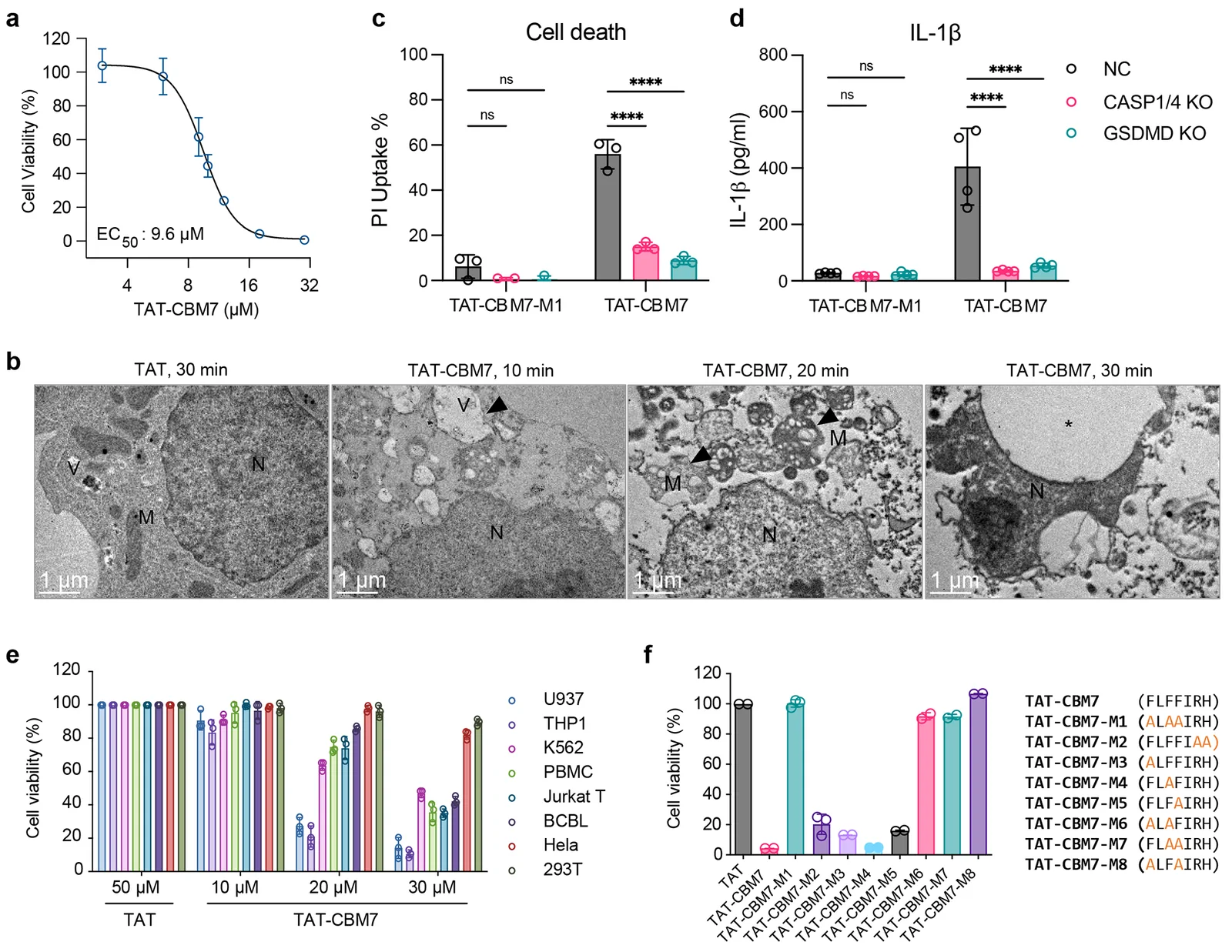
TAT-CBM7 triggers pyroptosis. **a**. Dose-response analysis of TAT-CBM7-induced cell death in U937 monocytes measured by ATP-based viability assay. **b**. TEM images of U937 cells treated with TAT-CBM7 or TAT at the indicated time points. N, nucleus; V, vacuole; M, mitochondria. **c-d**. Cell death and IL-1β secretion in WT (negative control, NC) or indicated knockout (KO) THP-1 macrophages treated with TAT-CBM7. Cell death was measured by propidium iodide uptake, and IL-1β was quantified by ELISA. Two-way ANOVA was performed for statistical significance. **e**. Cytotoxicity of TAT-CBM7 in different cell types following 20 min peptide treatment. **f**. Effects of CBM7 mutants on cell death post 20 min of treatment in U937 monocytes measured by ATP-based viability assay.

**Figure 8.**
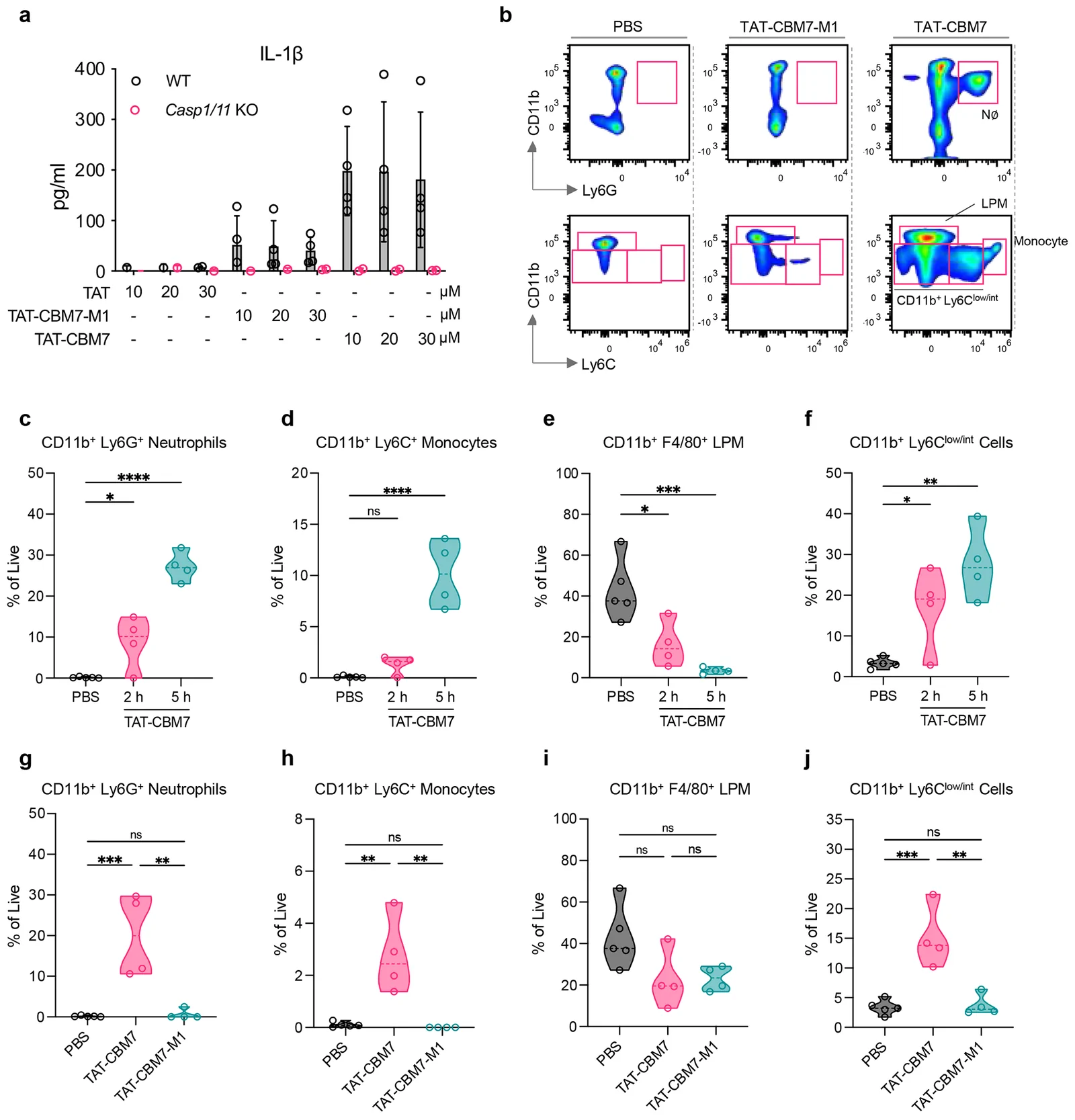
TAT-CBM7 promotes inflammation *in vivo*. **a**. IL-1β secretion from WT or *Casp1/11* KO BMDMs primed with LPS and treated with peptides. **b-j**. Flow-cytometry analysis of peritoneal immune-cell populations in C57BL/6 mice after intraperitoneal injection of peptides (n = 4) or PBS (n = 5). Representative gating plots were shown in panel (b). One-way ANOVA was performed for statistics.

## Data Availability

All data supporting the findings of this study, including the raw data underlying the figures, are provided as Source Data files with this paper.
